# Autophagy in renal fibrosis: Protection or promotion?

**DOI:** 10.3389/fphar.2022.963920

**Published:** 2022-08-24

**Authors:** Rong Dai, Lei Zhang, Hua Jin, Dong Wang, Meng Cheng, Tian Sang, Chuyi Peng, Yue Li, Yiping Wang

**Affiliations:** ^1^ Department of Chinese Medicine, Anhui University of Chinese Medicine, Hefei, China; ^2^ Department of Nephrology, the First Affiliated Hospital of Anhui University of Chinese Medicine, Hefei, China; ^3^ Graduate School, Anhui University of Chinese Medicine, Hefei, China; ^4^ Blood Purification Center, the First Affiliated Hospital of Anhui University of Chinese Medicine, Hefei, China

**Keywords:** autophagy, fibrosis, renal fibrosis, dual regulation, experimental model

## Abstract

Autophagy is a process that degrades endogenous cellular protein aggregates and damaged organelles via the lysosomal pathway to maintain cellular homeostasis and energy production. Baseline autophagy in the kidney, which serves as a quality control system, is essential for cellular metabolism and organelle homeostasis. Renal fibrosis is the ultimate pathological manifestation of progressive chronic kidney disease. In several experimental models of renal fibrosis, different time points, stimulus intensities, factors, and molecular mechanisms mediating the upregulation or downregulation of autophagy may have different effects on renal fibrosis. Autophagy occurring in a single lesion may also exert several distinct biological effects on renal fibrosis. Thus, whether autophagy prevents or facilitates renal fibrosis remains a complex and challenging question. This review explores the different effects of the dual regulatory function of autophagy on renal fibrosis in different renal fibrosis models, providing ideas for future work in related basic and clinical research.

## Introduction

Autophagy, first introduced in 1963 by Christian de Duve, is a Greek term meaning “self-eating” (Klionsky, 2008). From yeast to mammals, it is an evolutionarily conserved catabolic process by which some cytosolic components and organelles are transported to lysosomes for degradation and recycling (Mizushima et al., 2008). There are three types of autophagy in mammalian cells—macroautophagy, microautophagy, and molecular chaperone-mediated autophagy—which differ in the types of substrates degraded and in how they are transported to lysosomes. Macroautophagy (referred to here as autophagy), the focus of this paper, is the most classical and common type. It includes induction of nucleation, membrane extension and closure, formation of autophagic lysosomes, and degradation of the encapsulated contents. Microautophagy includes the direct phagocytosis of small cytoplasmic material in the lysosomal membrane invagination. Molecular chaperone-mediated autophagy is a process of direct transport of selected unfolded proteins across the lysosomal membrane for degradation via chaperone proteins (Levine and Kroemer, 2008; Mizushima et al., 2008; Ravikumar et al., 2010). The autophagy process consists of a series of cellular events. It is caused by the formation of a double-membrane cup-like structure, a phagosome, around an isolated cytoplasmic target site, followed by phagosome expansion and closure to form an autophagosome. The autophagosome then docks and fuses with the lysosome to form an autophagosome, in which the autophagosomal membrane and cytoplasmic matrix are degraded by acidic lysosomal hydrolases. Eventually, the resulting degradation products are released for recycling (Levine and Kroemer, 2008; Mizushima et al., 2008; Ravikumar et al., 2010; Mizushima and Komatsu, 2011).

**FIGURE 1:**
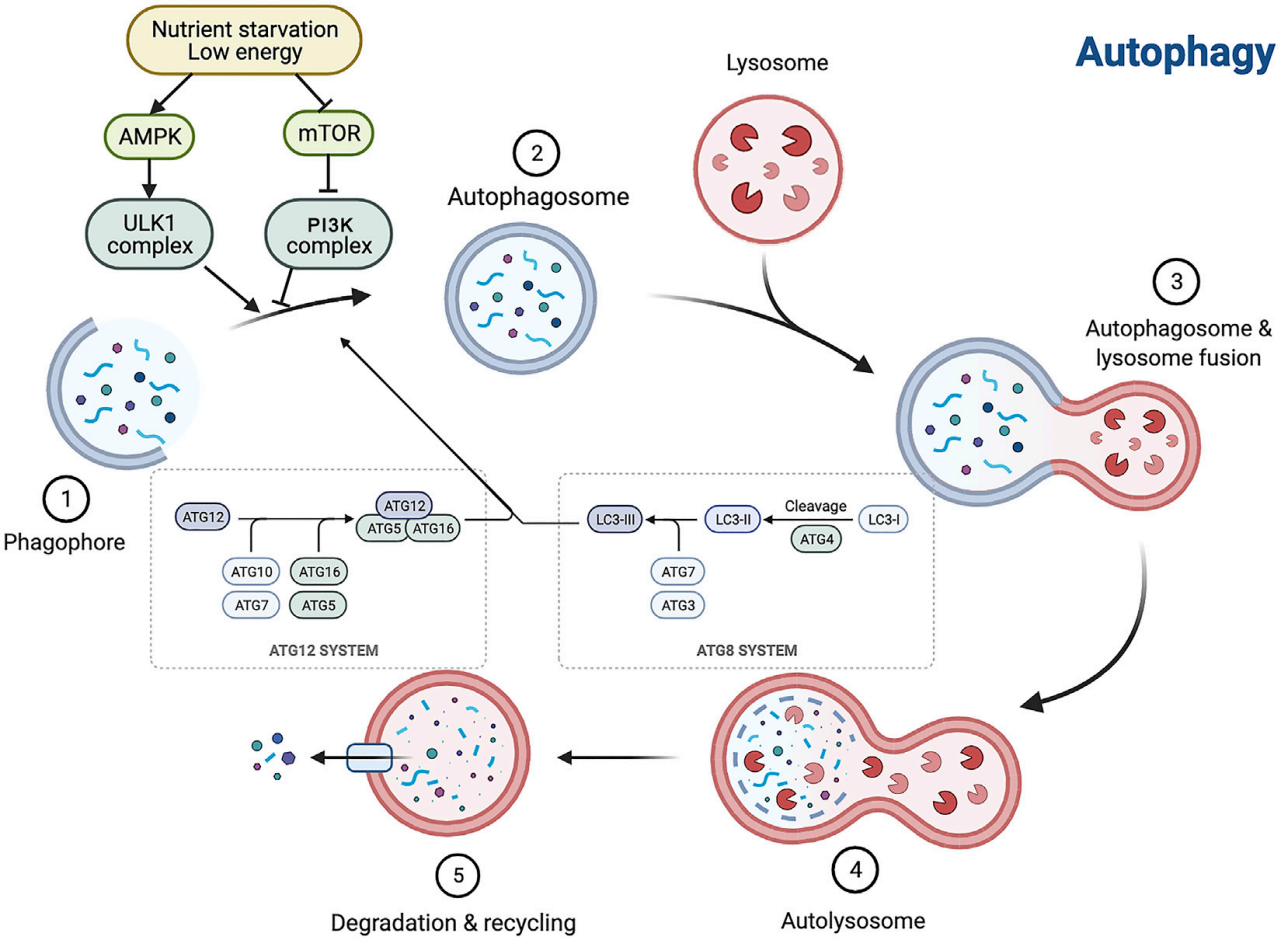
Autophagy is interfered with a broad variety of factors, and the autophagy process consists of a series of cellular events. Autophagy is a multistep process involving initiation, nucleation, expansion, fusion and degradation. AMPK, AMP-activated protein kinase; mTOR, mammalian target of rapamycin.

Autophagy is affected by a broad variety of factors, including extracellular factors such as nutrients in the outside world, ischemia and hypoxia, and concentration of growth factors, and intracellular factors such as metabolic stress, senescent or broken organelles, and misfolded or aggregated proteins. Because of the regular presence of these factors, cells maintain a low, basal autophagic activity to preserve self-stability. The level of autophagic activity is regulated by multiple signaling pathways, among which the mammalian target of the rapamycin (mTOR) pathway, AMP-activated protein kinase (AMPK) pathway, P53 pathway, acetylation modifications, phosphatidylinositol 3 kinase (PI3K)/protein kinase B (PKB, also known as AKT) pathway, and p62 play key roles. mTOR negatively regulates autophagy (Ravikumar et al., 2010; Inoki, 2014), and mTORC1 mainly regulates cell growth, apoptosis, energy metabolism, and cellular autophagy (Shaw, 2013; Mekahli et al., 2014). It can receive multiple signals of cellular changes and integrate them directly or indirectly through mTOR, thereby increasing or decreasing autophagy. AMPK inhibits mTOR kinase activity and activates autophagy by phosphorylating the TSC1/2 complex (Li and Chen, 2019). The P53 pathway is phosphorylated in response to stimuli such as nutrient deficiency or cytotoxicity, which in turn activates AMPK, inhibits mTOR, and activates autophagy (White, 2016). Acetylation is the addition of acetyl groups to the lysine or n-terminal fragments of target proteins by acetyltransferases; this process is involved in the regulation of autophagy initiation and selective autophagy by controlling the acetylation level of proteins important in the autophagic process (Wang and Wang, 2019). The PKB pathway activates PI3K and its downstream AKT, which in turn inhibits the TSC1/2 complex and activates mTOR, thereby inhibiting autophagy (Kaminskyy and Zhivotovsky, 2014). During autophagic vesicle formation, p62 acts as a bridge linking LC3 and ubiquitinated proteins, translocating abnormal proteins as receptors into autophagic vesicles, and degrading them through the ubiquitin signaling pathway (Jiang et al., 2015). Autophagy is regulated by several signaling pathways and related genes, and is closely related to the cell type and degree of cell differentiation; therefore, the specific biological effects of autophagy vary among different lesions but also even within a single lesion (Zhao et al., 2019; Kaushal et al., 2020) (Figure 1).

## Dual regulation phenomenon of autophagy

Numerous studies have shown that autophagy is widely present in cells of various tissues in the human body and is significant for maintaining normal physiological functions of the heart, liver, kidney, and other organs (Ueno and Komatsu, 2017; Sciarretta et al., 2018; Lin T. A. et al., 2019). Dysregulation or failure of autophagic pathways or mutations in autophagy-related genes can lead to a multitude of human diseases, including cancer, neurodegenerative diseases, chronic inflammatory diseases, and heart failure (Choi et al., 2013; Jiang and Mizushima, 2014; Galluzzi et al., 2017). Autophagy is induced in response to general cellular stress and pathological conditions, mainly as an adaptive and defensive strategy for cells to cope with survival stress (Sionov et al., 2015; Dikic and Elazar, 2018). Moreover, dysregulation of autophagy may contribute to the pathogenesis of various diseases, including alterations in factors regulating autophagy, duration, and intensity of autophagy in disease states (Huber et al., 2012; Choi et al., 2013). Programmed cell death protein 5 (PDCD5) is a cytosolic protein that activates the p53 pathway (Xu et al., 2012) . In PDCD5-overexpressing mice models, when subjected to acute stress overload, the ratio of LC3-II to LC3-I and the Beclin1 level significantly increases, which leads to upregulation of autophagy and a shift from stable cardiac hypertrophy to heart failure (Elgendy et al., 2011; An et al., 2012). Autophagy has a bidirectional role in preventing and exacerbating injury in the heart and in the liver. Protein overproduction due to Nrf2 and mTOR activation in the liver of autophagy-deficient mice may lead to hepatomegaly, inflammation, and liver tumorigenesis. Blocking of mTOR-mediated protein synthesis attenuates hepatomegaly and liver injury in young mice, but promotes early liver tumorigenesis. It is likely that the balance of autophagy is critical to maintaining normal liver physiology.

Therefore, autophagy has a dual role in preventing and exacerbating injury in most organs. In the kidney, autophagic activity is of great importance to maintain the stability, viability, and physiological function of the renal cellular internal environment (Inoki, 2014). In experimental models of renal fibrosis, autophagy has been shown to be a critical player in protecting renal function (Huber et al., 2012), but some studies have proved that autophagy activation can produce tissue damage (He et al., 2014; Mao and Zhang, 2015; Liu et al., 2016; Deng et al., 2017).

## Autophagy and fibrosis

Fibrosis is a phenomenon in which organs and tissues such as the lung, heart, liver, kidney, and skin show an increase in fibrous connective tissue and a decrease in parenchymal cells. Long-term fibrosis can lead to organ and tissue hypofunction and even functional failure. Autophagy is involved in the occurrence and development of fibrotic diseases. The effect of induced autophagy on fibrotic diseases has been demonstrated in most organs (Hernández-Gea et al., 2012). Reactive oxygen species are important downstream signaling factors for angiotensin II-mediated myocardial fibrosis; they can promote myocardial fibrosis by regulating TGF-β expression, which affects extracellular matrix (ECM) homeostasis and induces autophagy, thereby contributing to differentiation and proliferation of cardiac fibroblasts into myofibroblasts (Shi et al., 2015). Moreover, the regulatory role of autophagy in fibrotic diseases is bidirectional. For example, adipose mesenchymal stem cell–derived exosomes modified by miR-181-5p prevent liver fibrosis through autophagy activation (Qu et al., 2017). Nevertheless, miR-30a ameliorates liver fibrosis by inhibiting Beclin1-mediated autophagy (Chen et al., 2017). Kawarazin ameliorates Beclin1-induced pulmonary fibrosis by increasing miR-193a expression and inhibiting PI3K/Akt/mTOR signaling, thereby increasing autophagy in lung cells (Liu et al., 2019). IL-17A-producing T cells exacerbate fine particulate matter–induced pulmonary fibrosis by inhibiting PI3K/Akt/mTOR-mediated autophagy (Cong et al., 2020).

Renal fibrosis, characterized by excessive deposition of ECM in the glomerular and tubulointerstitial matrix, is a common pathological feature of progressive chronic kidney disease (CKD). The pathogenesis of renal fibrosis involves an extremely complex interplay of multiple cellular events, including fibroblast overproliferation and activation, increased ECM deposition, inflammatory cell infiltration, tubular atrophy, glomerulosclerosis, and sparse microvasculature (Liu, 2011; Duffield, 2014; Humphreys, 2018). It is now believed that renal fibrosis occurs mainly through epithelial–mesenchymal transition (EMT), activation of effector cells and local ischaemia and hypoxia, which affect various signalling pathways, ultimately leading to damage to the renal parenchyma and a decrease in glomerular filtration rate, thus progressing to chronic or end-stage renal disease (ESRD) (Gewin et al., 2017). The pathogenesis of renal fibrosis is complex, with multiple stimuli or mediators, including growth factors, cytokines, toxins and stress molecules, inducing fibrosis development through a variety of mechanisms and signalling (Falke et al., 2015; Zhang Z. H. et al., 2017).

**FIGURE 2:**
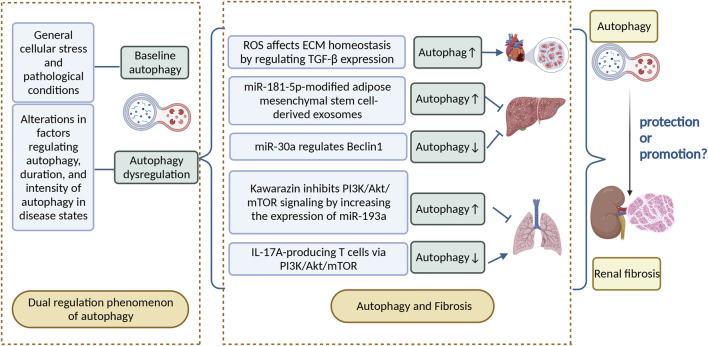
Autophagy can be divided into basal autophagy under physiological conditions and induced autophagy under stressful conditions. Autophagy has a bidirectional regulatory role in disease, as well as in fibrotic diseases, and the relationship between autophagy and renal fibrosis deserves further investigation. ROS, reactive oxygen species; ECM, extracellular matrix; IL-17A, interleukin-17.

The effect of autophagy on renal fibrosis varies in different tissues and settings, with some studies suggesting that autophagy has a protective effect on cells (Kawaoka et al., 2017) and others finding that excessive autophagy can damage the kidney (Inoue et al., 2010; Sansanwal et al., 2010). Given that autophagy may have a bidirectional regulatory effect on renal fibrosis, it should be examined whether the direction of the effect of autophagy in renal fibrosis depends on the degree of autophagy. As autophagy is a dynamic process, the boundary between protection and promotion against renal fibrosis is narrow, and likely depends on the time and intensity of autophagy induction and the molecular mechanism of autophagy activation and inhibition. In this paper, we review the process of the dual regulatory effects of autophagy from different models, aiming to discover specific and selective therapies to achieve effective regulation of autophagy, so as to provide theoretical guidance for the clinical prevention and delay of renal fibrosis progression (Figure 2).

## Models

### Autophagy in ischemia-reperfusion injury model

Acute kidney injury (AKI), caused mainly by nephrotoxic drugs, renal ischemia–reperfusion (IR), and sepsis, is a major renal disease with poor clinical outcomes in both the short term (high morbidity and mortality) and long term (development of CKD and ESRD) (Bellomo et al., 2012; Linkermann et al., 2014). The pathogenesis of AKI is multifactorial and involves complex interactions between microvascular, tubular, and inflammatory factors. Renal tubular cell injury and death are the main pathological features of this disease (Bellomo et al., 2012; Linkermann et al., 2014). AKI induced by IR (IR-AKI) is widely used as a model of AKI (Shiva et al., 2020); in this model, tubular inflammation and necrosis are seen initially, while persistent inflammation at a later stage may cause renal atrophy and increased renal fibrosis (Danelli et al., 2017). After IR injury, renal tubular cells have the ability to regenerate and repair, a process that involves the activation of multiple signaling pathways. Normal renal tubular repair begins with differentiation, migration, and proliferation of surviving cells to replace damaged cells, which is followed by re-differentiation to restore normal epithelial structure and function. However, renal tubular repair after severe or multiple AKI is often incomplete and maladaptive, leading to interstitial fibrosis and CKD (Ferenbach and Bonventre, 2015; Venkatachalam et al., 2015; Basile et al., 2016). Baseline autophagy in the kidney is essential for normal proximal tubule homeostasis, while autophagy activation has a protective role in IR injury (Jiang et al., 2010; Livingston and Dong, 2014). However, at different stages of the disease, defective autophagy in renal tubular cells may also promote cell proliferation and contribute to tubular regeneration and repair, which in turn improve renal fibrosis (Kaushal and Shah, 2016).

In both *in vivo* and *in vitro* models, autophagy occurs as a response to IR injury (Chien et al., 2007; Suzuki et al., 2008; Isaka et al., 2009; Kimura et al., 2011; Turkmen et al., 2011; Liu L. et al., 2012). A number of studies have demonstrated the beneficial role of autophagy in the process of IR injury. In specific autophagy-related gene 5 (ATG5) knockout mice, which have autophagy defects in proximal tubules, autophagy-deficient cells aggregate deformed mitochondria and cytoplasmic inclusions, leading to cellular hypertrophy and eventual degeneration, which has not been observed in wild-type controls. In autophagy-deficient mice, IR injury increases proximal tubule apoptosis and accumulation of p62 and ubiquitin-positive cytoplasmic inclusions. Serum urea nitrogen and creatinine are significantly elevated in autophagy-deficient mice compared with control animals (Kimura et al., 2011). This acts as evidence identifying that autophagy can maintain the stability of the internal environment of proximal tubule cells and prevent ischemic injury, and enhanced autophagy may attenuate the progression of AKI and renal fibrosis (Kimura et al., 2011).

Currently, drug-induced autophagy activation provides evidence for a protective role of autophagy in IR injury. For instance, vitamin D has potential nephroprotective effects. Specifically, treatment of a rat model of IR injury with 22-oxocalciferol (OCT), a synthetic vitamin D analogue, has revealed that OCT + IR-injured rats exhibit significantly increased autophagy and reduced cell cycle block compared with IR-injured rats. Moreover, OCT may induce autophagy through the Janus kinase/signal transducer and activator of transcription (JAK/STAT) pathway–induced phosphorylation of the Bcl-2–Beclin-1 complex to activate autophagy (Huhtakangas et al., 2017), attenuate IR injury–induced deterioration of AKI function and histological damage, and significantly reduce renal fibrosis (Hamzawy et al., 2019). In addition, rapid reperfusion of ischemic kidneys is effective in restoring renal function, reducing post-ischemic renal damage, and reversing the progression of renal fibrosis (Forbes et al., 2000). Ischemic postconditioning (IPO) was originally proposed by Zhao et al. to reduce the extent of injury by repeated transient arterial blockade in the early stages of reperfusion (Zhao et al., 2003). Previous studies have demonstrated the protective effect of IPO on renal fibrosis after IR injury (Weng et al., 2012; Zhang S. et al., 2017). Autophagy was later found to be involved in the protective effect of IPO against IR injury. There is increasing evidence suggesting that IPO significantly reduces renal tubular epithelial cell apoptosis and improves renal function on day 2 after IR injury, and autophagy is significantly activated in the kidneys of IPO rats. Two months after reperfusion, IR-injured rats exhibit severe renal fibrosis and EMT, and renal fibrosis and EMT are significantly reduced in IPO-treated rats. It has been shown that the levels of TGF-β1 and α-SMA are much lower in the IPO-treated kidneys than in the IR group. Furthermore, waveform protein expression is increased and e-calmodulin expression is decreased in the IR group. All these findings also indicate that the IPO is able to improve renal fibrosis by enhancing the activation of autophagy and inhibiting the extent of EMT after IR injury (Song et al., 2018). In summary, the lack of autophagy during IR injury leads to an increase in the degree of kidney damage, and the repair of IR injury is associated with an increase in autophagic activity. Nevertheless, unilateral renal IR injury (uIR) contributes to induction of renal endoplasmic reticulum stress (ERS), activation of autophagy in renal tubular cells, macrophage infiltration, and an increase in the proinflammatory factors MCP-1, IL-6, and TNF-α. Administration of tauroyl deoxycholic acid (TUDCA) and 4-phenylbutyric acid (4-PBA) block ERS in the post-ischemic kidney and inhibit tubular autophagy and renal fibrosis, indicating a profibrotic effect of autophagy after IR injury (Shu et al., 2018). What is the role of autophagy in renal fibrosis due to ischaemic injury in the kidney? Does it protect or promote? This remains a complex question.

A recent study assessed autophagic activity during IR injury and recovery phases. Li et al. generated CAG-RFP-EGFP-LC3 mice under chicken b-actin control using the differential sensitivity of red fluorescent protein (RFP; pKa 4.5) and enhanced green fluorescent protein (EGFP; pKa 5.9) to pH, so as to distinguish between early autophagic vesicles and autolysosome (Kimura et al., 2007; Zhou et al., 2012). Because RFP is stable at an acidic pH and enhanced GFP is quenched at an acidic pH, the formation of acidic autolysosomes during IR injury is distinct from that of autophagosomes. In this study, basal enhanced GFP and RFP fluorescence did not change when examined 45 min after ischemia or 4 h after reperfusion, indicating that autophagy was not activated during this time. In the late phase of reperfusion, enhanced GFP and RFP fluorescence peaked 24 h after reperfusion, but only RFP fluorescence persisted 3 days after reperfusion. Moreover, RFP fluorescence returned to basal levels 7 days after reperfusion. These studies revealed dynamic changes in renal tubular autophagy after IR injury and during recovery by showing that autophagosome formation decreased 24 h after reperfusion, and autophagy lysosome formation persisted during renal recovery to clear autophagosome formation. Mechanistically, mTORC1 is activated after IR injury to inhibit autophagy by preventing the formation of Atg complexes. mTORC1 activation may prevent autophagy initiation or lead to autophagy breakdown by accelerating the fusion of autophagosomes and lysosomes or enhancing the degradation of autophagosome content (Li et al., 2014). It has been suggested that the activation of autophagy in the early stage of IR injury helps delay the progression of renal fibrosis, while the disappearance of autophagy in tubular cells during the recovery period may promote tubular regeneration and repair, thus improving renal fibrosis. Hence, the effect of autophagy on renal fibrosis is a dynamic process.

Normal EPO/EpoR signaling in renal tubules is involved in the regulation of tubular autophagic flux; high EpoR activity in the kidney reduces AKI in the acute phase; and low EpoR activity makes IR injury more likely in the acute phase. Upregulation of EPO/EpoR signaling pathway in the early phase can reduce renal injury, and downregulation of EPO/EpoR signaling after the acute phase can reduce autophagic flux, maintain autophagy–apoptosis balance and peritubular capillary integrity, and promote recovery from renal injury, thereby inhibiting renal fibrosis (Shi et al., 2018). These results reflect that the effect of autophagy on renal fibrosis is related to the period of disease onset, and attention should be paid to the dynamic regulation of autophagy to maximize the effect of delaying renal fibrosis. Of note, controlling the degree of autophagy activation is critical for the regulation of renal fibrosis.

In IR injury models, autophagy enhances cell survival during initial tubular injury as a response to AKI, but hinders normal repair during reperfusion. The long-term pro-fibrotic effect of autophagy may be due to its cytoprotective role. Activation of autophagy in response to AKI helps delay the progression of renal fibrosis in most cases, while defective autophagy during the recovery period after injury may promote renal tubular cell reparation to improve renal fibrosis. In addition, the specific timing and extent of autophagy activation after IR injury may either promote or prevent the progression of renal fibrosis; the specific regulation depends on the activation factors and the different signaling pathways (Table 1).

**TABLE 1:** Dual regulation of autophagy in ischemia-reperfusion injury model.

Intervention factors	Mechanism	Effect on autophagy	Effect on fibrosis	Citation
OCT (22-oxocalciferol)	Activation of autophagy by phosphorylation of the Bcl-2–Beclin-1 complex induced by the Janus kinase/signal transducer and activator of transcription (JAK/STAT) pathway	↑	↓	Hamzawy et al. (2019)
IPO (Ischemic preconditioning)	Extent of EMT inhibition after IR injury	↑	↓	Zhang et al. (2017a)
ERS (Endoplasmic reticulum stress)	Macrophage infiltration, increase in MCP-1, IL-6, and TNF-α proinflammatory factors	↑	↑	Shu et al. (2018)
ATG5 knockout	Increased proximal tubule apoptosis and accumulation of p62 and ubiquitin-positive cytoplasmic inclusions	↓	↑	Kimura et al. (2011)
CAG-RFP-EGFP-LC3	mTORC1 is activated after IR injury and inhibits autophagy by preventing the formation of Atg complexes	↑/↓	↑	Li et al. (2014)
EPO/EpoR	Damage to peritubular capillary structures and induction of renal tubular hypoxia	↑/↓	↑	Shi et al. (2018)

### Autophagy in unilateral ureteral obstruction model

To date, most studies on the role of autophagy in renal interstitial fibrosis have been performed using the unilateral ureteral obstruction (UUO) model. UUO is currently a more classical animal model of renal interstitial fibrosis (Chevalier et al., 2009), which can respond to inflammatory cell infiltration, tubular cell apoptosis and necrosis, transdifferentiation of mesangial cells to fibroblasts, fibroblast aggregation, ECM deposition, and tubular atrophy, among a series of pathological changes (Li et al., 2010; Kim W. Y. et al., 2012). In the UUO renal fibrosis model, autophagy also plays two crucial roles: in protecting and a role in promoting renal fibrosis.

Several studies have proposed that autophagy has antifibrotic effects in UUO-associated renal fibrosis. Autophagy decreases ECM deposition and renal tubular atrophy, thereby reducing the extent of renal fibrosis (Kim W. Y. et al., 2012; Ding and Choi, 2014). Valproic acid (VPA) is a histone deacetylase (HDAC) inhibitor, which has been shown to induce autophagy (Göttlicher et al., 2001; Blaheta and Cinatl, 2002; Fu et al., 2010). VPA may improve renal fibrosis by inducing autophagy, while 3-MA increases renal fibrosis and inhibits autophagy. In UUO mice, 3-MA decreases the antifibrotic effect of VPA. Renal fibrosis is a state of excessive ECM protein production, and in previous studies VPA may have induced autophagy and thus ameliorated renal fibrosis by promoting ECM and degradation (Strutz and Zeisberg, 2006; LeBleu et al., 2013). Autophagy can also exhibit its effects on the process of renal fibrosis by regulating the TGF-β1 and NLRP3 inflammatory vesicle signaling pathways. Ding et al. demonstrated the role of autophagy in promoting mature TGF-β1 degradation in UUO kidney and TGF-β1-treated renal tubular epithelial cells (RTECs), further implying that autophagy can inhibit renal interstitial fibrosis through negative regulation of TGF-β1 (Ding et al., 2014). Noninflammatory vesicle-dependent NLRP3 in renal tubular cells plays a critical role in mitochondrial ROS production and damage after hypoxic injury via posthypoxic MAVS relocalization. The mitochondrial regulation in the absence of NLRP3 increases autophagy and attenuates apoptosis after UUO, which in turn prevents the progression of renal fibrosis (Kim et al., 2018). Sphingosine kinase 1 (SK1) converts sphingosine into endogenous sphingosine-1-phosphate (S1P), which regulates autophagy and is involved in the course of fibrotic disease (Rojas-Canales et al., 2017). Knockdown of SK1 leads to decreased autophagy, while overexpression of SK1 leads to a higher degree of increased autophagy (Lavieu et al., 2006; Pyne et al., 2016). In the UUO model, both SK1 enzyme activity and the autophagic response are upregulated. Moreover, there is increased expression of mature TGF-β1 and increased ECM deposition in the tubulointerstitial region of the kidney compared with sham-operated mice. It has been reported that SK1 expression induces autophagy activation and protects the kidney during fibrosis (Du et al., 2017a). Collectively, these study finding support the notion that activation of autophagy, in most cases, inhibits renal fibrosis in the obstructed kidney and may provide a pro-survival effect. However, activation of autophagy may also contribute to exacerbation of renal fibrosis.

Interestingly, the activation of autophagy in UUO model renal interstitial of myofibroblasts promotes the progression of fibrosis. Protein kinase Cα (PKCα) is one of the major subpathways of mTORC2, and the Rictor/mTORC2 signaling pathway is involved in TGFβ1-induced fibroblast activation and renal fibrosis (Li et al., 2015). Previous studies have identified that PKCα signaling is activated in mesenchymal myofibroblasts in fibrotic kidneys of UUO mice, which in turn mediates TGF-β1-induced fibroblast activation and promotes renal fibrosis by promoting autophagic flux (Xue et al., 2018). As autophagic activity is controlled by autophagosome formation and autophagosome degradation, the rate of autophagic turnover is defined as the autophagic flux (Zhang et al., 2013). These results suggest that an increase in autophagic flux is associated with an increase in renal fibrosis.

In addition, recent studies have revealed a profibrotic role of autophagy downregulation or defect in UUO-associated renal interstitial fibrosis. Using a proximal tubule-specific ATG5 knockout mice model, Li et al. proposed that autophagy deficiency promotes G2/M cell cycle block and accelerates renal interstitial fibrosis after UUO (Li et al., 2016). A recent study provided strong evidence that phosphatase and tensin homolog (PTEN)-induced kinase 1/(PINK1)/mitotic fusion 2 (MFN2)/Parkin–mediated mitotic phagocytosis of macrophages is downregulated during renal fibrosis, and deletion of PINK1 or Parkin promotes the progression of macrophages to mitotic/M2 macrophages, which favors renal fibrosis (Bhatia et al., 2019). The adaptive response of cells is closely linked to the cell cycle (Thomasova and Anders, 2015). Inhibition of autophagy can cause cells to remain in the G2/M phase and increase ECM deposition, which in turn exacerbates renal fibrosis. Enhancing autophagy, from another perspective, prevents cells from staying in the G2/M phase, which in turn reduces ECM deposition (Li et al., 2016). This implies that inhibition of autophagy leads to metabolic derangement of cells and thus promotes fibrous progression, that there is a close link between the cell cycle and renal tissue injury, and autophagy can delay renal tissue injury by regulating the cell cycle.

In contrast, autophagy downregulation or deficiency in the UUO model can also attenuate renal fibrosis. In exploring the antifibrotic effects of G-Rb1 in the G-Rb1 UUO mice model, it has been shown that G-Rb1 reverses UUO-induced p62 downregulation, LC3 upregulation, and LC3 I/II ratio, suggesting that G-Rb1 inhibits UUO-induced autophagy activation and thus attenuates renal fibrosis (Liu et al., 2020). Tang et al. used the Gene Expression Omnibus database (GEO) to extract and analyze miRNAs and mRNAs that may be associated with RF in UUO model mice (Tang et al., 2020). The bioinformatics analysis showed that PTCH1 expression was regulated by miR-342-5p and FoxO3. The results showed that PTCH1 expression was regulated negatively by miR-342-5p and positively by FOXO3, PTCH1-induced autophagy in TCMK-1 cells stimulated by TGF-β1 was downregulated and renal fibrosis was ameliorated (Tang et al., 2020). JNK-associated leucine zipper protein (JLP) is a potential endogenous antifibrotic factor. JLP is mainly expressed in normal human or mouse RTECs, and its expression is downregulated in renal fibrosis. In UUO mice, JLP deficiency results in more severe renal fibrosis, whereas renal fibrosis resistance is observed in RTECs-specific transgenic mice. JLP plays a protective role in the negative regulation of TGF-β1 expression and autophagy in renal fibrosis, as well as in the profibrotic effects of ECM production, EMT, apoptosis, and cell cycle block in RTECs (Yan et al., 2020). Itaconic acid is an endogenous metabolite with anti-inflammatory and antioxidant effects. Itaconic acid 4-octyl ester (OI), a competitor of itaconic acid, has high lipid solubility, penetrates cell membranes, and is metabolized to itaconic acid *in vitro*. OI ameliorates renal fibrosis by inhibiting activation of the TGF-β/Smad and nuclear factor kappa-light-chain-enhancer of activated B cells (NF-κB) pathways, reducing ROS production and inhibiting autophagy (Tian et al., 2020). Upregulation of TGF-β1 plays a central role in the pathogenesis of renal fibrosis, and it is now well accepted that TGF-β1 induces autophagy in thylakoid cells, thereby negatively regulating matrix production through the degradation of intracellular type I collagen. Therefore, TGF-β1 is both an inducer of collagen synthesis and an inducer of autophagy and subsequent collagen degradation. Indeed, TGF-β1 can also mediate PI3K/Akt activation of the mTOR pathway, which may have both activating and inhibiting effects on autophagy, which may depend on the specific cell type and environment (Zhang et al., 2021).

Different methods of autophagy induction have different effects on the regulation of renal fibrosis. In a study by Xu et al., a renal fibrosis model was established in Rab7 knockout mice (prepared by CRISPR/Cas9 technology) and wild-type (WT) C57BL/6 mice UUO. Rab7 is involved in the formation and transport of autophagosomes and subsequent fusion with lysosomes (Ao et al., 2014; Guerra and Bucci, 2016). The expression of Rab7 tended to increase over time in WT mice. Moreover, autophagic activity increased continuously in both groups of mice, although it was higher in Rab7 knockout mice than in WT mice at the same time point. Renal fibrosis was less severe in Rab7 knockout mice than in WT mice 7 days after UUO, but became more severe 14 days after surgery. Thus, different autophagy induction methods can produce different physical responses that may either delay or lead to more severe renal fibrosis. The extent of fibrosis in Rab7 knockout mice may be associated with autophagy regulation of lipophagy and endocytosis. For example, UUO-induced intracellular lipid accumulation in renal tubular cells is significantly reduced when the pharmacological inhibitors 3-MA or CQ are administered concomitantly, while renal interstitial fibrosis, tubular cell apoptosis, and tubular cell dedifferentiation are attenuated. Some parts of overdeposited ECM and inflammatory cells are phagocytosed and degraded, thereby delaying the progression of renal fibrosis in mice. The continued activation of autophagic activity at a later stage results in lipid deposition in the kidney and decreased ECM degradation, which lead to more severe renal fibrosis (Xu et al., 2020). This fibrosis-associated lipid accumulation is independent of the phagocytic-lysosomal pathway, but it is dependent on Beclin1. These results suggest a role for autophagy in regulating lipid metabolism in renal tubular cells. A recent study by Yan et al. has further confirmed the connection between sustained activation of autophagy and lipid accumulation in renal tubular epithelial cells during renal fibrosis (Yan et al., 2018). During starvation and malnutrition, autophagy needs to be induced to degrade lipids and provide energy for the whole body to maintain normal activity (Minami et al., 2017). However, UUO-induced autophagy contributes to excessive lipid deposition, and this lipotoxicity exacerbates renal fibrosis (Livingston et al., 2016). Thus, autophagy may be involved in lipid metabolism and thus bidirectional regulation of renal fibrosis through inducing lipolysis and promoting lipid accumulation to induce lipotoxicity in renal tubular cells.

Overall, in UUO-induced renal fibrosis, the improvement of fibrosis by upregulation of autophagy is mainly manifested by the inhibition of inflammatory signals during fibrosis, degradation of the cellular matrix and regulation of the cell cycle by autophagy. The pro-fibrotic effect of autophagy is mainly due to the shrinkage of tubules and lipid accumulation caused by sustained activation. Differences in the mode of induction to autophagy and the timing of autophagy activation, the molecular mechanisms mediated and the signal pathways, result in a possible dual role of autophagy in the regulation of renal fibrosis. Sustained activation of autophagy may lead to tubular atrophy and thus promote renal fibrosis, whereas autophagy-mediated degradation of excess collagen may prevent fibrosis (Table 2).

**TABLE 2:** Dual regulation of autophagy in unilateral ureteral obstruction model.

Intervention factors	Mechanism	Effect on autophagy	Effect on fibrosis	Citation
VPA (valproic acid)	Promotion of ECM protein and degradation, induction of autophagy	↑	↓	(Strutz and Zeisberg, 2006; LeBleu et al., 2013)
RTECs	Negative regulation of TGF-β1	↑	↓	Ding et al. (2014)
NLRP3	Playing an important role in the production and damage of mitochondrial ROS after hypoxic injury	↑	↓	Kim et al. (2018)
SK1 (sphingosine kinase 1)	Increased expression of mature TGF-β1 in the renal tubular interstitial region and increased ECM deposition	↑	↓	Du et al. (2017a)
G-Rb1	Trans-UUO-induced p62 downregulation, LC3 upregulation, and LC3 I/II ratio	↑	↓	Liu et al. (2020)
PTCH1	Negatively regulated by miR-342-5p and positively regulated by FOXO3	↑	↓	Tang et al. (2020)
JLP	Negative regulation of TGF-β1 expression	↑	↓	Yan et al. (2020)
Rab7	Involved in autophagosome formation and transport, and subsequent fusion with lysosomes	↑	↑	(Ao et al., 2014; Guerra and Bucci, 2016)
3-MA or CQ	Significantly reduced intracellular lipid accumulation in renal tubular cells, as well as reduced interstitial	↑	↑	Xu et al. (2020)
PKCα (protein kinase Cα)	Mediation of TGF-β1-induced fibroblast activation	↑	↑	Xue et al. (2018)
ATG5 knockout	Cell cycle G2/M blockade	↓	↑	Li et al. (2016)
PINK1/Parkin	Promoted the development of macrophages to mitotic/M2 macrophages	↓	↑	Bhatia et al. (2019)

### Autophagy in diabetic kidney disease model

Diabetic kidney disease (DKD) is a glomerular disease, and tubulointerstitial fibrosis (TIF) always occurs early in DKD (Podgórski et al., 2019). TIF is prominent in glomerular disease, which progresses to chronic renal failure. TIF is also characterized by tubular atrophy and excessive accumulation of ECM components. Furthermore, early accumulation of renal ECM in patients with diabetes usually leads to glomerulosclerosis and interstitial fibrosis (Huang F. et al., 2019). Classical DKD models are streptozotocin (STZ)-induced, Akita, NOD, or obese type 2 diabetes models (Azushima et al., 2018). In DKD, autophagy may be of great importance in stopping or exacerbating the progression of the disease process through its regulatory role on renal fibrosis.

Recent studies have confirmed that activation of autophagy can inhibit fibrosis and halt DKD progression (Huang H. et al., 2019). In P2Y2R deficiency, inhibitory phosphorylation of ULK-1 is reduced and downstream Beclin-1 autophagic signaling is activated (Russell et al., 2013). Increased expression of SIRT-1 and FOXO3a in mice with P2Y2R deficiency enhances the autophagic response, which ameliorates renal interstitial fibrosis in mice with DKD (Dusabimana et al., 2020). Klotho has originally been identified as an antiaging factor (Barbosa Júnior Ade et al., 1992). It is mainly expressed in the kidney, especially in distal renal tubular cells (Kuro-o, 2009). Klotho overexpression significantly reinforces autophagy, AMPK and ERK1/2 activity *in vitro* and *in vivo*, which can be abrogated by selective AMPK inhibitors and ERK activators. It has been suggested that Klotho exhibits its effects in renal protection by activating autophagy in renal tubular cells through AMPK and ERK pathways and participating in the fibrotic process (Xue et al., 2021). *Cyclocarya paliurus* (CP) is an herbal plant from China. Triterpenic acids-enriched fraction from CP (CPT) increases AMPK phosphorylation and decreases phosphorylation of its downstream effector mTOR, which in turn activates autophagy and improves thylakoid stromal fibrosis (Zhang et al., 2019). The aforementioned study showed that activation of autophagy through the AMPK and ERK pathways contributes to ameliorate renal fibrosis, and additionally a similar phenomenon was found in AKT/mTOR. Oleanolic acid (OA), a potential drug for DKD, has been shown to increase autophagy by regulating miR-142-5p/PTEN through inhibition of the PI3K/AKT/mTOR pathway, thereby decreasing interstitial fibrosis (Chen et al., 2019). It is well documented that KCa3.1 is widely expressed throughout the body, and KCa3.1 regulates Ca^2+^ entry and modulates Ca^2+^ signaling in these cells. It is known that elevated intracellular Ca^2+^ inhibits autophagy (Reidy et al., 2014). Elevated intracellular Ca^2+^ maintains increased mTORC1 activity through a pathway independent of AMPK (Devi et al., 2012). Because KCa3.1 regulates Ca^2+^ entry, there is an interaction between KCa3.1 and autophagy. In DKD models, it has been shown that blocking KCa3.1 reverses diabetes-inhibited tubular autophagy and thus improves renal fibrosis, which is mediated by inhibiting the activation of the PI3K/Akt/mTOR signaling pathway (Huang et al., 2016). Triptolide (TP), a traditional Chinese medicine, increases autophagy via the miR-141-3p/PTEN/Akt/mTOR pathway in a high-fat diet (HFD)-fed rat STZ-induced DKD model, thereby reducing fibrosis (Li et al., 2017).

Conversely, several studies have shown that inhibition of autophagy attenuates renal fibrosis. LncRNA SOX2OT overexpression attenuates the pathogenesis of DKD by reducing Akt/mTOR-mediated autophagy and significantly inhibiting thylakoid cell proliferation and fibrosis (Chen et al., 2021). Therefore, in DKD models, although the pathways mediating autophagy are the same, differences in the induction factors may also result in activation or inhibition of autophagy, which in turn exerts a mitigating effect on renal fibrosis. The interaction between autophagy and the AMPK signaling pathway, PI3K/AKT/mTOR pathway, and other signaling pathways further illustrates that autophagy is a complex life activity. Hence, a single regulatory outcome mechanism cannot accomplish such a complex regulation, and the organism maintains intracellular autophagy in a normal and orderly manner through a complex regulatory network. The complex interactions between autophagy and multiple signaling molecules indicate that the regulatory mechanism of a single signaling pathway can hardly explain the transformation of the intracellular autophagy level and should be considered synergistically from multiple targets. Inhibition of autophagy may exacerbate renal fibrosis. In the STZ-induced IL-17A knockout (KO) DKD mice model, IL-17A-KO STZ-treated mice develop more severe renal injury, with increased glomerular injury and interstitial fibrosis at week 12 (Tan et al., 2022). IL-17A deficiency also increases the upregulation of proinflammatory cytokine and fibrosis gene expression after STZ treatment. Compared with WT mice, IL-17A-KO-STZ-treated mice have significantly lower levels of LC3 and ATG7, which play a key role in autophagosome formation. Thus, these findings highlight that IL-17 deficiency exacerbates STZ-induced interstitial fibrosis by attenuating the autophagic response (Kim et al., 2021). These studies demonstrate that enhanced autophagy of fibrosis in the thylakoid region and interstitium during DKD fibrosis promotes the progression of fibrosis. Autophagy may exacerbate renal fibrosis by increasing inflammation.

In DKD models, microRNAs may be involved in the activation or inhibition of autophagy, which in turn has different outcomes on renal fibrosis. In normal rat kidney (NRK)-52E cells, overexpression of miR-22 inhibits autophagic flux and induces expression of Col IV and α-SMA, leading to increased renal fibrosis (Zhang et al., 2018). Under high glucose conditions, a concentration gradient of miR-155 is detected in HK-2 cells, with increased p53 expression and downregulated expression of SIRT1 and autophagy-associated proteins LC3II, ATG5, and ATG7 (Zhang et al., 2019). miR-155 can target its binding to the SIRT1 3′ UTR region to reduce its expression. Overexpression of miR-155 reduces LC3-II and ATG5 expression in HK-2 cells under high glucose conditions. Inhibition of the signaling axis of p53, miR-155-5p, and SIRT1 activates autophagy, which may serve as a protective response against renal fibrosis cell survival and ameliorate diabetic kidney injury (Zhang et al., 2019). Autophagy impairment in DKD is associated with downregulation of unc-51-like autophagy-activated kinase 1 (ULK1), and ULK1 can upregulate miR-214 in diabetic kidney cells and tissues. Knockdown of miR-214 from the proximal tubule of the kidney prevents reduced ULK1 expression and autophagic injury in diabetic kidneys, and the blockade of p53 attenuates the induction of miR-214 in DKD, leading to higher levels of ULK1 and autophagy, while improving renal interstitial fibrosis (Ma et al., 2020). Thus, the same type of factors may contribute to both activation and inhibition of autophagy, which in turn produces a bidirectional regulation of renal interstitial fibrosis.

In summary, autophagic integrity is critical for cellular homeostasis and its alteration can lead to cellular damage or death. DKD is associated with severe dysregulation of autophagy. In DKD models with the same pathways mediating autophagy, differences in the inducing factors may result in activation or inhibition of autophagy, which in turn exerts a mitigating effect on renal fibrosis. Furthermore, the same type of factor-induced autophagy may bring about activation or inhibition of autophagy owing to differences in the molecular mechanisms and signaling pathways that mediate autophagy. Therefore, we may infer that the regulatory role of autophagy in renal fibrosis may be bidirectional in the DKD model (Table 3).

**TABLE 3:** Dual regulation of autophagy in diabetic kidney disease model.

Intervention factors	Mechanism	Effect on autophagy	Effect on fibrosis	Citation
Klotho	Enhanced AMPK and ERK1/2 activity	↑	↓	Xue et al. (2021)
CPT (triterpenic acids-enriched fraction from CP)	Increased phosphorylation of AMPK and decreased phosphorylation of its downstream effector mTOR	↑	↓	Zhang et al. (2019)
OA (oleanolic acid)	Inhibition of PI3K/AKT/mTOR pathway regulates miR-142-5p/PTEN	↑	↓	Chen et al. (2019)
KCa3.1	LC3 and nitrotyrosine expression and phosphorylation of mTOR were significantly increased	↑	↓	Reidy et al. (2014)
TP (triptolide)	miR-141-3p/PTEN/Akt/mTOR pathway	↑	↓	Li et al. (2017)
miR-155	Inhibition of the signaling axis of p53, miR-155-5p, and SIRT1	↑	↓	Zhang et al. (2019)
miR-214	Resulting in higher levels of ULK1	↑	↓	Ma et al. (2020)
lncRNA SOX2OT	Reduction of Akt/mTOR-mediated autophagy	↓	↓	Chen et al. (2021)
OI (Itaconic acid 4-octyl ester)	Inhibition of TGF-β/Smad and NF-κB pathways	↓	↓	Tian et al. (2020)
IL-17A-KO	LC3 and ATG7 levels were significantly reduced	↓	↑	Kim et al. (2021)
miR-22	Induces expression of Col IV and α-SMA	↓	↑	Zhang et al. (2018)

### Autophagy in other models

The IR injury model, the UUO model, and the DKD model are the most widely used in experimental models of renal fibrosis. The regulation of autophagy shows a bidirectional effect on renal fibrosis, both in these three models and in other models. Activation of autophagy in AKI has initially been demonstrated in an experimental model of cisplatin-induced nephrotoxicity (Periyasamy-Thandavan et al., 2008; Yang et al., 2008). Retinoic acid (RA) is a major derivative of vitamin A. RA protects RTECs from cisplatin-induced injury, activates autophagy, inhibits cisplatin-induced apoptosis, attenuates cisplatin-induced tubular injury, and reduces inflammation and fibrosis in renal injury (Wu et al., 2020). Autophagy has a critical effect on regulating the inflammatory response of renal tissues (Du et al., 2017b). It exerts a blocking, protective effect by reducing the local inflammatory response and inhibiting the release of inflammatory factors. However, the relationship between renal fibrosis and the inflammatory response is complex and close; the inflammatory response has its own distinct biological effects on glomeruli and tubules; and the exact biological effects of autophagy remain to be investigated. Transcription factor EB (TFEB) is a major regulator of autophagy, and related experiments have shown that TFEB-mediated activation of autophagy may give rise to autophagic cell death and inflammation in renal tubular epithelial cells, resulting in adenine-induced renal fibrosis in CKD (Yuan et al., 2021). The NLRP3 inhibitor dapansutrile (DAPA) has shown promising efficacy in various inflammatory diseases after clinical phase II trials. DAPA reduces the autophagy marker LC-3 and attenuates the exacerbation of folate-induced renal fibrosis by targeting the inflammasome/cystein-1/IL axis (He et al., 2014). Koesters et al. used a tetracycline-controlled mouse model and showed TGF-β1-specific overexpression in renal tubules, revealing that sustained expression of TGF-β1 promoted tubular autophagy, thereby leading to tubular dedifferentiation and extensive peritubular fibrosis. Notably, such degenerating cells are not positive for apoptosis in TUNEL-stained pores, revealing that autophagy may be a key driver of tubular atrophy in TGF-β1-induced renal fibrosis (Koesters et al., 2010). In mice models of sepsis, autophagy activation by the mTORC1 inhibitor tesilomorph or the AMPK activator 5-aminoimidazole-4-carboxamide ribonucleotide (AICAR) is protective against the development of sepsis-associated AKI and delays the progression of renal fibrosis (Wang et al., 2019). A recent study by Brooks et al. further revealed a novel epithelial biological mechanism that integrates phagocytosis, autophagy, and antigen presentation to the regulation of the inflammatory response after injury (Brooks et al., 2015). Kidney injury molecule-1 (KIM-1) is expressed on proximal renal tubular cells and converts cells into phagocytes to take up tubular apoptotic cell debris (Ichimura et al., 2008). KIM-1-mediated phagocytosis is subsequently processed by autophagy to efficiently remove apoptotic cells and autophagic degradation of phagosomes, leading to major histocompatibility complex (MHC) restriction of antigen presentation and inhibition of CD4^+^ T cell proliferation, and increases the percentage of regulatory T cells in an autophagy gene-dependent manner. These results emphasize the role of autophagy in reducing the post-injury inflammatory response and maintaining proximal tubular cell self-tolerance, both of which contribute to the amelioration of renal fibrosis (Brooks et al., 2015). Rhubarb and its bioactive component, rhubarbic acid, are frequently used in East Asian countries for the treatment of CKD. It is acknowledged that in a model of adenine-induced tubular injury in rats, where autophagy activation is accompanied by renal fibrosis, rhubarb acid can inhibit autophagy by modulating the AMPK-dependent mTOR signaling pathway and key molecules in ERK and p38 MAPKs signaling pathways, thereby reducing renal fibrosis (Tu et al., 2017). It has been suggested that the glucagon-like peptide-1 (GLP-1) analogue liraglutide (LRG) enhances autophagy to reduce lipid accumulation in hepatocytes involved in the AMPK/mTOR pathway (He et al., 2016). Therefore, we hypothesized that GLP-1 and its receptor GLP-1R are associated with autophagy and suggested that GLP-1 or GLP-1R may be effective targets for autophagy-related diseases. It was found that LRG could activate autophagy through AMPK/mTOR signaling in a 5/6 nephrectomy rat model, thereby attenuating renal injury (Xue et al., 2019).

In summary, in the adenine-induced CKD model, the folic acid-induced renal fibrosis model, the tetracycline-induced renal fibrosis model, the inhibition of autophagy attenuated the level of renal fibrosis, while the enhancement of autophagy in the cisplatin-induced tubular injury model and the septic renal fibrosis model attenuated the level of renal fibrosis. In different models of renal fibrosis, the same or similar potent biological effects may be induced, leading to activation or inhibition of autophagy, which in turn exacerbates or attenuates the level of renal fibrosis. Whether this difference in inducing injury factors is the main factor causing the difference in the role of autophagy expression in different models deserves further attention (Table 4).

**TABLE 4:** Dual regulation of autophagy in other models.

Intervention factors	Mechanism	Effect on autophagy	Effect on fibrosis	Citation
RA (retinoic acid)	Mitigated cisplatin-induced tubular injury and reduced inflammation in renal injury	↑	↓	Wu et al. (2020)
TFEB (transcription factor EB)	Autophagic cell death and inflammation in renal tubular epithelial cells	↑	↓	Yuan et al. (2021)
Tesilomorph or AICAR	mTORC1 inhibition or AMPK activation	↑	↓	Wang et al. (2019)
KIM-1 (Kidney injury molecule-1)	Increase in percentage of regulatory T cells in an autophagy gene-dependent manner	↑	↓	Brooks et al. (2015)
liraglutide (LRG)	Activate autophagy through AMPK/mTOR signaling	↑	↓	He et al. (2016)
DAPA (dapansutrile)	Targeting the inflammasome/cystein-1/IL axis	↓	↓	He et al. (2014)
Rhubarb acid	Regulation of AMPK-dependent mTOR signaling pathway and key molecules in ERK and p38 MAPKs signaling pathways	↓	↓	Tu et al. (2017)
TGF-β1	Renal tubular dedifferentiation	↑	↑	Koesters et al. (2010)

## Cell autophagy and renal fibrosis

Autophagy is an important mechanism for maintaining cellular homeostasis in all major types of renal intrinsic cells, including RTEC, glomerular mesangial cells (GMC), podocytes and glomerular endothelial cells (GEC). Renal tubular epithelial cell injury is a central event in the pathogenesis of CKD (Zhou et al., 2019). RECT autophagy is protective in maintaining RTEC integrity under physiological, AKI or aging conditions (Kimura et al., 2011; Liu S. et al., 2012; Lin Q. et al., 2019), but its role in renal fibrosis remains controversial. G2/M blockade of RTEC after renal injury is associated with increased cytokine production. It has been demonstrated that ATG5-mediated autophagy in proximal tubular epithelial cells attenuates G2/M cell cycle block and inhibits renal fibrosis (Li et al., 2016). These results suggest that RTEC autophagy may prevent renal fibrosis by inhibiting G2/M phase block. Furthermore, *in vitro* studies, ATG5 attenuated the inflammatory response by inhibiting NF-κB signaling, which is dependent on the functional role of autophagy. This finding provides important insights into the mechanisms by which ATG5 inhibits renal inflammation and highlights the importance of autophagy in the role of renal protection. Manipulation of autophagy may be a novel therapeutic approach for inflammatory nephropathy (Peng et al., 2019). However, it has also been shown that sustained activation of autophagy in RTEC promotes renal fibrosis by regulating interstitial inflammation and the secretion of pro-fibrotic factors (Livingston et al., 2016). Using an *in vitro* model of TGF-β-induced HK-2 cells, Yan et al. found that silencing Beclin1, a gene upstream of autophagy in HK-2 cells, reduced TGF-β-induced lipid deposition, suggesting that autophagy promotes lipid droplet formation in renal tubular epithelial cells in a Beclin1-dependent manner, leading to renal lipotoxicity and promoting progression of renal fibrosis (Yan et al., 2018). These results suggest that the role of RTEC autophagy in renal fibrosis remains inexact and it may produce opposite results due to differences in high and low autophagic activity. Similarly, GMC autophagy may play a bidirectional role in regulating renal fibrosis. Ding et al. found that under serum deprivation conditions, TGF-β induced GMC autophagy through TAK1-MKK3-P38 and PI3K/Akt-dependent pathways, enhancing cell survival by inhibiting thylakoid apoptosis (Ding et al., 2010). On the other hand, activated autophagy is involved in the intracellular degradation of type I collagen, which inhibits renal fibrosis by negatively regulating and preventing the continued accumulation of ECM (Kim S. I. et al., 2012). These results demonstrate that GMC autophagy may act as an adaptive mechanism in response to glomerular injury by inhibiting GMC apoptosis and promoting their survival. However, recently Lee et al. found that chrysin inhibited renal fibrosis by suppressing the mTOR pathway and inhibiting the induction of GMC autophagy-related genes (Lee et al., 2019). Therefore, the role and mechanism of GMC autophagy in renal fibrosis remains unclear and deserves further investigation.

Autophagy plays an important role in maintaining podocyte homeostasis, and its dysfunction may be associated with the renal fibrosis process. Clinical and experimental evidence suggests that dysfunction of the autophagy-lysosome pathway leads to severe podocyte injury, podocyte loss, massive proteinuria, and renal fibrosis (Oshima et al., 2011; Cinà et al., 2012). In addition, GEC autophagy plays an important role in maintaining GEC integrity, podocyte ultrastructure, glomerular filtration barrier (GFB) homeostasis, and glomerular capillary integrity (Satchell and Braet, 2009; Jourde-Chiche et al., 2019). Notably, altered autophagy genes on the other side of the GFB involving endothelial-specific ATG5 deletion also lead to capillary thinning and accelerated DKD. These data suggest that autophagy is a key protective mechanism for both cell layers of the GFB, suggesting that GEC and podocyte autophagy synergistically protect against renal fibrosis (Lenoir et al., 2015). In addition, current studies have focused on restoring the level of podocyte autophagy by regulating mTOR upstream of autophagy, thereby reducing glomerulosclerosis (Chen et al., 2005). Over-activation of the mTOR pathway in DKD plays a key role in the process of podocyte injury and decreased glomerular filtration rate. Damaged podocytes are always accompanied by reduced autophagic flux, accumulation of p62, and interaction of p62 with LC3 (Komatsu et al., 2007). In high glucose exposed MPC5 cells, activation of mTOR and enhanced p62 expression and LC3II/LC3I rates were significantly inhibited, suggesting adipose-derived stem cells-Exo mediated increase in autophagic flux in damaged podocytes. These results also support that regulation of autophagic homeostasis in podocytes contributes to amelioration of renal fibrosis (Jin et al., 2019). Therefore, induction of autophagy in podocytes and GEC may reduce the extent of renal fibrosis to some extent, but the exact molecular mechanisms remain to be explored in depth.

Through the above study we found that both high or low autophagy in RTEC and GMC may play a role in protecting renal fibrosis. Knockdown of different autophagy genes, or defective autophagy in different segments of RTEC, may lead to different renal fibrosis outcomes. However, autophagy in podocytes and GEC plays a predominantly protective role in maintaining cellular integrity and inhibiting renal fibrosis. They do so mainly through intercellular synergism, modulation of relevant signaling pathways, and regulation of relevant cellular processes. In addition, there are fewer studies related to the role of autophagy in other kidney resident cells as well as in infiltrating cells in promoting or inhibiting renal fibrosis.

## Conclusion and perspectives

**FIGURE 3:**
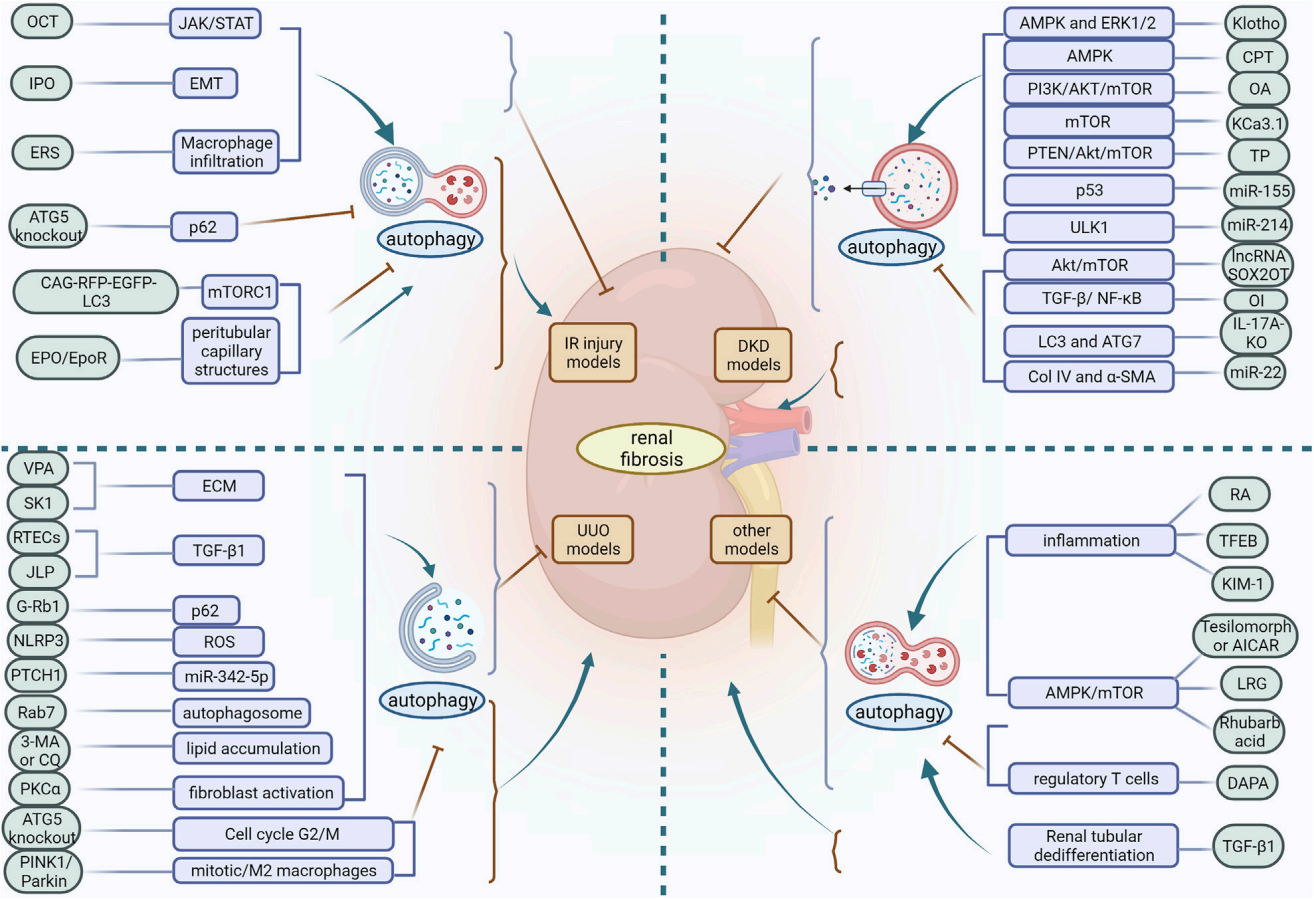
In different experimental models of renal fibrosis, autophagy has a dual regulatory role. OCT, 22-oxocalciferol; IPO, Ischemic postconditioning; ERS, endoplasmic reticulum stress; VPA, Valproic acid; SK1, Sphingosine kinase 1; ECM, extracellular matrix; RETCs, renal tubular epithelial cells; JLP, JNK-associated leucine zipper protein; ROS, reactive oxygen species; PKCα, protein kinase Cα; CPT, triterpenic acids-enriched fraction from CP; OA, oleanolic acid; TP, triptolide; OI, Itaconic acid 4-octyl ester; RA, retinoic acid; TFEB, transcription factor EB; KIM-1, Kidney injury molecule-1; LRG, liraglutide; DAPA, dapansutrile.

Autophagy is an evolutionarily conserved degradation process whose basal level plays a critical role in the stability of the internal environment. The role of autophagy in the pathogenesis of renal disease may be multifactorial and complex, and its biological effects can differ considerably depending on the pathological location of the diseased tissue. From the above findings discussed in this review, it is clear that autophagy is a double-edged sword. Based on *in vivo* models of acute and chronic kidney injury, the role of autophagy in renal fibrosis is multifaceted. Depending on the experimental conditions, autophagy may be protective or detrimental. In IR injury models, activation of autophagy slows the progression of renal fibrosis in most cases, but defective autophagy later in the course of the disease may contribute to ameliorate renal fibrosis. In the UUO model, differences in how autophagy is induced also produce different regulatory outcomes in renal fibrosis. In the DKD model, different inducers of the same pathway may activate or inhibit autophagy and exert a mitigating effect on renal fibrosis. Moreover, in different experimental models of renal fibrosis, differences in injury methods, cell types, the timing and intensity of autophagy induction, and the diversity of signaling molecular mechanisms lead to contradictory effects of autophagy on renal fibrosis (Figure 3). Therefore, differences in the factors regulating autophagy should be carefully considered in order to maximise its protective effect. Furthermore, the function of autophagy in most cells and its impact on renal fibrosis remains poorly understood. Autophagy in podocytes and GEC plays a protective role in maintaining cellular integrity and inhibiting renal fibrosis. However, the role of RTEC and GMC autophagy in renal fibrosis remains controversial, with some studies suggesting that RTEC and GMC autophagy can inhibit renal fibrosis while others suggest the opposite. Apart from these studies in knockout mice, there is no strong evidence that autophagy in other renal resident cells, as well as in infiltrating cells, plays a role in promoting or inhibiting renal fibrosis.

A large body of evidence supports an important role for autophagy in renal physiology and pathology. Despite these advances, many unanswered questions remain. For example, while studies have now reported negative regulation between autophagy and some classical pathways in the kidney, whether there is positive regulation remains to be further explored. It is well known that autophagy plays different roles in different diseases or stages of the same disease, and whether the differential mediating autophagy triggers play different roles on renal fibrosis in different periods of renal disease, such as early, middle and late stages, needs to be further elucidated. In addition, at this stage, some animal and cellular experimental studies have demonstrated that drugs can reduce renal fibrosis by regulating autophagy, but whether they can be extended to clinical treatment remains open. In many cases, autophagy may interact with other cellular processes to influence the development of renal disease, and it is also important to investigate such interactions and the regulatory mechanisms involved. Additionally, the evidence supporting autophagy malfunction in renal illnesses that is now available comes from hypothesis-driven research and is still absent from unbiased studies like omics research.

Autophagy is a dynamic process, and it is difficult for a single regulatory mechanism to complete this complex process, and the organism works through a complex regulatory network to maintain orderly intracellular autophagy. Therefore, the complex connection between autophagy and multiple signaling molecules should be considered synergistically from multiple targets. At present, it is not clear how autophagy affects renal fibrosis through signaling molecule interactions in a dynamic equilibrium. It is believed that through more detailed studies of the autophagic process in the future, multi-targeted intervention of autophagic signaling pathways, intermittent control of autophagic activation time and intensity, and constant changes of autophagy-inducing factors can be achieved to control autophagy in a moderate range, and targeted autophagy will become a new strategy for delaying renal fibrosis.
